# Use of the Vascularized Iliac-Crest Flap in Musculoskeletal Lesions

**DOI:** 10.1155/2013/237146

**Published:** 2013-10-22

**Authors:** Cristiane Tonoli, Alexandre H. S. Bechara, Roberto Rossanez, William D. Belangero, Bruno Livani

**Affiliations:** Orthopaedic Department of HC-UNICAMP, P.O. Box 6142, 13083-888 Campinas, SP, Brazil

## Abstract

Bone loss was in the past treated by several methods, such as bone distraction and the use of nonvascularized or tissue-bank bone grafts. With the advent of modern microsurgical techniques, the vascularized bone flap has been used with good results; it resolves local nutritional problems, repairs soft tissue that is often damaged by severe trauma, and treats bone loss due to tumors, pseudarthroses, and osteomyelitis. This paper reports the authors' experience with the use of vascularized iliac-crest flaps to treat orthopedic pathologies in five patients with traumatic bone loss (<10 cm), three with osteomyelitis, and three with atrophic nonunion. In all cases, the same surgeon obtained a vascularized iliac-crest flap with a pedicle based on the deep iliac circumflex artery. All flaps consolidated within a mean period of 3 months. These findings demonstrate that the use of an iliac-crest flap is a treatment option in cases of bone loss and that it is associated with good functional results and minimal donor-site morbidity.

## 1. Introduction

Reconstructive orthopedic surgery to treat bone loss due to severe trauma, pseudarthroses, chronic osteomyelitis, or tumors can be performed using a range of techniques, such as external or internal fixation and the use of endoprostheses or nonvascularized bone flaps [1–3]. However, these techniques do not resolve the vascularization problems that can occur in these morbid conditions. With the development of the microsurgical technique, the vascularized bone flap has proven to be a good reconstructive option because it enables greater bone integration, higher consolidation rates, and better functional response [1, 4, 5].

The nonvascularized bone graft has been used with good results in cases with <6 cm bone loss [2]. The vascularized fibula flap has achieved good functional results in defects >12 cm [3], although some authors have reported high rates of donor-site morbidity, such as weakness (37%), flexion/extension difficulty (29–43%), flexion contractions, residual ankle pain, and fibular nerve lesions [6, 7]. In cases with intermediate amounts (5–10 cm) of bone loss, the use of the vascularized iliac-crest flap retains the benefits of a vascularized graft without incurring late donor-site morbidity [8]. Although iliac-crest flaps >12 cm have been used, they are not recommended because fractures may occur due to the stress applied to the lower limb [9]. Given the evidence of the benefits of vascularized iliac-crest flap application in cases of moderate bone loss, this flap was used to treat 11 patients with orthopedic lesions of different etiologies.

## 2. Patients and Methods

After obtaining institutional ethics committee approval, we retrospectively identified all patients who had been treated with vascularized iliac-crest grafts between January 2009 and January 2012. The inclusion criteria were acute or chronic bone loss secondary to trauma or other bone lesions, need for graft-associated osteosynthesis, such as in cases of atrophic pseudarthrosis, or pseudarthrosis due to osteomyelitis. Exclusion criteria were prior trauma in the affected region, prior pathology, such as rheumatoid arthritis or peripheral vascular insufficiency (which could affect local vascularization and graft success), psychiatric or mental disorders, neurological damage, and institutionalization.

All qualifying medical charts were analyzed, and the subjects were asked to return as outpatients. Those who agreed to participate in the study were asked to provide written informed consent (legal representatives provided consent for patients <18 years of age). Medical records were examined, and radiographs were evaluated separately by two orthopedic surgeons. Both were part of the study but did not participate in the surgery and did not know the patients (blind study). 

Eleven patients were treated with vascularized iliac-crest flaps between January 2009 and January 2012. The etiology of bone loss (4–8 cm) was a motorcycle accident in cases  1–5 (Table 1). Two patients had bone loss in the wrist (distal radius), two patients in the femur (distal femoral metaphysis), and one patient in the foot (first metatarsal). One patient (case  11) had bone loss in the left radius secondary to atrophic nonunion of a fracture treated with osteosynthesis and a dynamic compression plate (DCP). The other cases had no bone loss but atrophic nonunion of the right humerus (case  7) or right radius (case  9) or nonunion secondary to osteomyelitis with a history of previous osteosynthesis in the left forearm (case  6), the right forearm (case 8), or the left humerus (case  10) (Table 1).

All surgeries were performed by a single experienced surgeon according to the technique described in the literature [9–11].

Each patient was placed in the supine position with the donor site raised. A skin incision was made from the location of the anterior superior iliac spine toward that of the inguinal ligament. The pedicle was based on the deep iliac circumflex artery, which emerges from the external oblique muscle adjacent to the superior border of the iliac crest. The oblique external and internal muscles were freed, and the pedicle was found between the transverse and oblique internal muscles. It was then dissected along the inguinal ligament to the external iliac artery, where the pedicle originates 2 cm above the ligament [12]. The deep iliac circumflex artery may originate from the femoral artery (42.2% of cases) or from the external iliac artery below (40.6%) or above (17.2%) the inguinal ligament [11]. The external diameter of the artery is 1–5 mm at its origin, and the distance to the anterior superior iliac spine is 6–27 (mean: 15.8 ± 5.6) mm. 

The receptor site was then prepared, the flap was removed by osteotomy with a saw, and the arterial pedicle was removed after ensuring that it was sufficiently large (4–6 cm) to enable anastomosis [12]. After hemostasis, the donor site was sutured.

 At the anastomosis site, 2000 units of heparin were administered intravenously. No heparin was used postoperatively, but oral acetyl salicylic acid (100 mg) was prescribed for 30 days.

Osteosynthesis was performed at the receptor site using DCPs and screws or Kirschner wires (case  4). Continuity of the medullar canal was reestablished in all cases. Consolidation was verified clinically and radiologically. Clinical criteria included absence of pain, lack of mobility at the fracture site, and return to daily activities (work and leisure). The radiological criteria included absence of implant failure (signs of loosening or breakage), signs of graft incorporation and bone fracture healing, continuity of the trabecular bone through the bone fragments and graft, presence of bone callus, and presence of at least three out of four cortices in the anteroposterior and lateral radiographs [13].

## 3. Results

**Figure 1:**
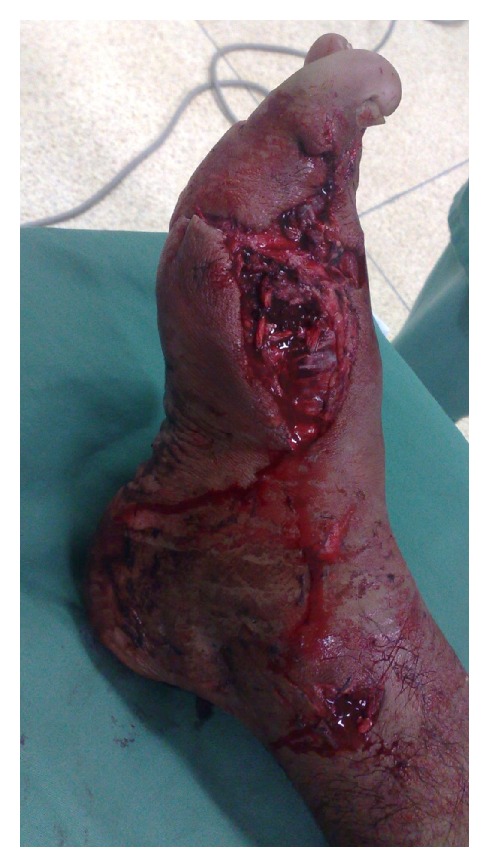
Case 4—soft-tissue loss.

**Figure 2:**
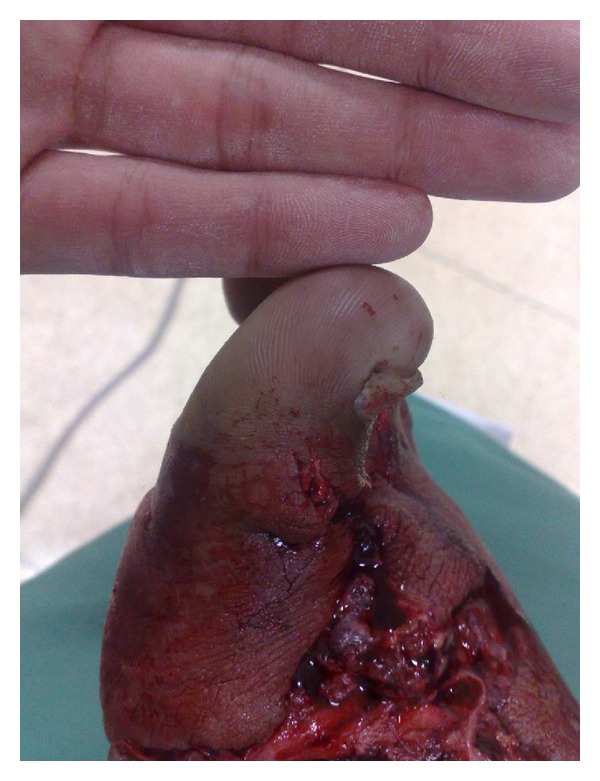
Case 4—reduced perfusion.

**Figure 3:**
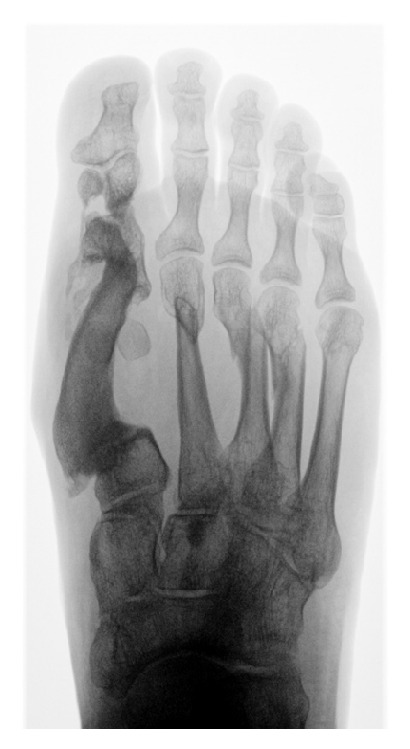
Case 4—final radiograph of consolidated bone graft.

**Figure 4:**
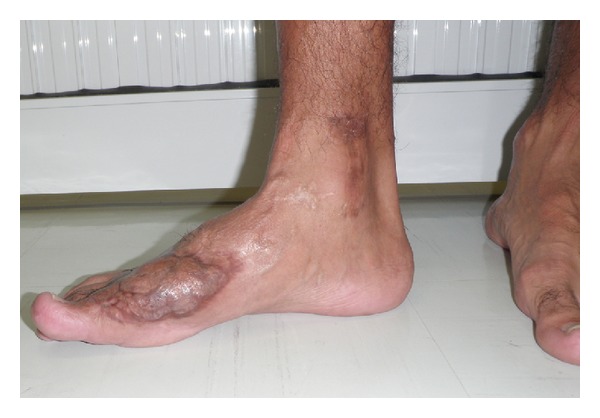
Case 4—final clinical result.

All flaps consolidated within a mean period of 3 months. Soft tissue and musculature adjacent to the iliac crest, which shared an arterial supply with the flap, were transferred with the graft in case  4  (Figure 1). The distal perfusion deficit (Figure 2) was recovered by the same procedure with good results (Figures 3 and 4).

In case  6, consolidation occurred rapidly (within 4 weeks) because the patient was a child, but a purulent fistula persisted, necessitating a second procedure to remove the plate and provide local debridement.

In many of the cases reported here, the iliac-crest flap was used to treat lesions that were due to high-energy trauma; this flap was also used to treat other serious fractures and nerve or vascular lesions, including traumatic amputations (case  10, four fingers) and late amputations (case  8, right leg amputation after vascular graft failure) (Table 2).

The follow-up period ranged from 7 to 36 months. All patients reported postoperative pain at the donor site, which was intense in the first postsurgical days but disappeared after 4 weeks. Common analgesics were used to relieve pain. Late morbidity (contour irregularity of the basin) occurred in case  11.

## 4. Discussion

Several traditionally used bone reconstruction techniques yield limited results when bone loss is associated with disruption of the local blood supply. This complex situation is commonly encountered in cases of high-energy trauma, chronic osteomyelitis, pseudarthrosis, and tumor resection, and the only feasible solution is often amputation. The use of a vascularized bone graft resolves issues related to bone nutrition and permits simultaneous soft-tissue reconstruction [14, 15].

The advantages of the vascularized bone flap over the conventional bone graft are better osseointegration, higher consolidation rates, and improved functional response [1, 4, 5]. Hence, in cases of severe bone loss or in situations in which other procedures have yielded poor results, the vascularized bone flap is a better option than the nonvascularized graft because it provides more tissue viability, in terms of vascular nutrition, during the bone healing process. Due to this improved local blood supply, bone production, and intrinsic stability, the vascularized flap has demonstrated faster bone consolidation and a better functional outcome [1, 3, 5, 13, 14, 16].

The vascularized iliac-crest flap is superior to the fibula flap for the treatment of small defects because its size enables better adaptation and because donor-site morbidities, such as ankle rigidity or instability and fibular nerve lesions, can be prevented [17, 18].

The selection of the best donor site and the choice of a vascularized or conventional bone graft are multifactorial processes that must take into account not only the defect size, but also the compatibility between donor site and receptor regions, structural features of the graft (cortical, spongy, or osteomuscular), mechanical demands of the defect site, duration of the procedure, and donor-site morbidity. Therefore, even in cases with <6 cm of bone loss, the vascularized bone graft is often the best option, especially for defects in the extremities (hands and feet) that demand greater mechanical freedom and precision of movement. In a study of ankle arthritis due to massive bone loss secondary to tumors, osteomyelitis, and trauma, Bishop et al. [6] reported that iliac-crest flaps were appropriate for defects <4 cm. The vascularized iliac-crest flap has also been used successfully for reconstruction of the maxilla and mandible (97%) [9], distal radius [12], and carpals [19] and for the treatment of ankle instability [20].

The vascularized fibular flap has shown good results in the treatment of defects with ≤26 cm bone loss [15]. The complication rates are low (11.5%) [7] and include contracture of hallux flexion (4.3%), ankle pain (4.1%), weakness (0.6%), and fibular nerve lesions (1.7%). Only a small percentage (13%) of patients with weakness and ankle instability (37%) [6] demonstrated weakness during a physical examination. Other complications include ankle stiffness, cellulitis, suture dehiscence [17, 18], and ankle valgus deformity [15, 21]. Rogers et al. [22] found no significant difference in donor-site morbidity between vascularized iliac-crest and fibula flaps. Although the removal of the iliac vascular graft is a more complicated procedure than the removal of the nonvascularized tricortical graft, the potential complications at the donor site are almost the same: hematoma, infection, postoperative pain, lesion of the lateral femoral cutaneous nerve, and the Trendelenburg gait. However, when bone loss is more than 6.0 cm, other options of vascularized bone grafts such as the scapula and fibula also present potential morbidity at the donorsite [22–24]. It is possible to use the iliac-crest flap in gaps from 6 cm to 10 cm and up to 12 cm. For greater bone loss, the vascularized fibula should be considered. Moreover, fibular graft is a strut graft therefore more resistent, but iliac graft as a cancelous bone graft has a higher healing potential than fibular strut graft [9].

The vascularized iliac-crest flap requires practice in microsurgical techniques as it is a complex procedure and is only indicated in cases where bone loss is more than 5 to 6 cm. However, the results obtained using nonvascularized grafts in these conditions are not as good [4, 8, 25].

Although postoperative pain was the main donor-site morbidity in the present study, others have reported cellulitis (3.6%), deep vein thrombosis (3.6%), and femoral nerve neuropraxia (4.8%) as early complications. The main late complications reported are changes in anterolateral thigh sensitivity (27%), contour irregularity of the basin (20%), and hernias (9.7%) [23, 24].

The vascularized iliac-crest flap is an option for the treatment of bone loss, especially in small bones. Its advantages include a long pedicle (4–6 cm) and large diameter, which facilitate anastomosis [9]. Moreover, in cases of concomitant soft-tissue loss and perfusion deficit, this flap enables the transfer of muscle and skin covering the iliac crest because these tissues are supplied by the same artery (case  4, Figures 1 and 2). This option is also useful in cases of severe nonunion in which a conventional graft has failed. Furthermore, in cases of anastomosis failure, the iliac crest functions better than a long bone as a conventional graft due to its corticocancellous component.

The limitations of this study include its retrospective design and small sample size. The cases presented is heterogeneous because bone loss in orthopedic traumatology cases is relatively rare and so are the indications for the use of the vascularized flap. The recipient bed must have a rich vascular supply, which in some cases of severe trauma and/or osteomyelitis/pseudarthrosis is not possible due to the presence of vascular injury secondary to these illnesses. Therefore, it is difficult to find similar bone-loss cases with an indication for a vascularized flap. Moreover, most of the patients are cases of high-energy trauma, a variable population depending on the circumstances of the trauma [8, 9]. Cases of bone loss are relatively rare and most often are severe cases with multiple fractures resulting from trauma, nonunion, or difficult-to-treat osteomyelitis. Therefore, the number is small as they are cases of major morbidity. However, in the case of locomotor trauma, the series reported using the vascularized iliac-crest flap is not small when compared to the literature [8, 20]. This flap is mostly used in head [22], neck, and maxillofacial surgery [9, 17].

Because some traumatic lesions were treated directly with the vascularized bone graft, comparison with other types of treatment was not possible. However, the findings indicate that the vascularized iliac-crest flap is a reliable and safe treatment option that yields good results and is associated with minimal donor-site morbidity in cases of bone and muscle loss secondary to trauma and osteomyelitis.

## Figures and Tables

**Table 1:** Clinical information.

Patient	Age (years)	Sex	Previous diagnosis	Location	Amount of bone loss	Etiology of bone lesion (flap receptor)
Case 1	55	M	Open fracture with bone loss in distal radius	Left radius	6 cm	Trauma
Case 2	30	F	Fracture of distal radius with median nerve injury	Right radius	5 cm	Trauma
Case 3	25	F	Open fracture of femur with bone loss	Left femur	8 cm	Trauma
Case 4	23	M	Open fracture of foot with a lesion and loss of soft tissue	Right first metatarsal	6 cm	Trauma
Case 5	34	M	Open fracture	Left femur	8 cm	Trauma
Case 6	09	M	Closed fracture of forearm that developed osteomyelitis after osteosynthesis with Kirschner wires	Left radius	—	Nonunion, secondary to osteomyelitis
Case 7	66	M	Closed fracture treated with immobilization plaster	Right humeral diaphysis	—	Atrophic nonunion (conservative treatment)
Case 8	24	F	Closed forearm fracture that developed osteomyelitis after osteosynthesis with DCP	Right radius	—	Nonunion, secondary to osteomyelitis
Case 9	56	M	Closed fracture that developed atrophic nonunion of the radius after osteosynthesis with DCP	Right radius	—	Atrophic nonunion
Case 10	31	M	Open fracture that developed osteomyelitis and nonunion after osteosynthesis with DCP	Left humerus	—	Nonunion, secondary to osteomyelitis
Case 11	40	F	Closed fracture that developed atrophic nonunion of the radius after osteosynthesis with DCP	Right radius	5 cm	Atrophic nonunion

M: male; F: female; DCP: dynamic compression plate.

**Table 2:** Casuistry.

Patient	Consolidation time (months)	Follow-up period (months)	Complications	Type of synthesis	Duration of postoperative pain at donor site (days)	Associated lesions
Case 1	1.5	36	—	2 plates	5	—
Case 2	2	30	—	2 plates	8	Median nerve lesion (trauma)
Case 3	2.5	24	—	2 plates	9	Closed femoral neck fracture and open femoral and tibial diaphysis fractures (all ipsilateral)
Case 4	2	17	—	Kirschner wires	10	Medial malleolus fracture (ipsilateral)
Case 5	3	7	—	2 plates	14	Closed tibial plateau fracture (ipsilateral) and closed right distal radius and forearm fractures
Case 6	1	7	Persistent infection requiring second procedure	1 plate	8	—
Case 7	4	8	—	1 plate	10	—
Case 8	3	16	—	1 plate	15	Right sacroiliac joint lesion, left ischiopubic branch fracture, right femur and tibia open fractures, popliteal artery vascular injury and vascular graft failure after 2 days requiring leg amputation
Case 9	3	12	—	Third semitubular plate	21	—
Case 10	4	21	—	1 LCP	21	Brachial plexus injury (ipsilateral) + traumatic amputation of fingers 2–5
Case 11	3	12	Contour irregularity of basin	1 DCP (radius) + third semitubular plate (ulna)	30	—

LCP: locking compression plate; DCP: dynamic compression plate.
